# Engineering Chlorophyll, Bacteriochlorophyll, and Carotenoid Biosynthetic Pathways in *Escherichia coli*

**DOI:** 10.1021/acssynbio.3c00237

**Published:** 2023-08-02

**Authors:** Guangyu E. Chen, C. Neil Hunter

**Affiliations:** † State Key Laboratory of Microbial Metabolism, School of Life Sciences and Biotechnology, Shanghai Jiao Tong University, Shanghai 200240, China; ‡ School of Biosciences, University of Sheffield, Sheffield S10 2TN, United Kingdom

**Keywords:** photosynthesis, chlorophyll, bacteriochlorophyll, carotenoid, biosynthesis

## Abstract

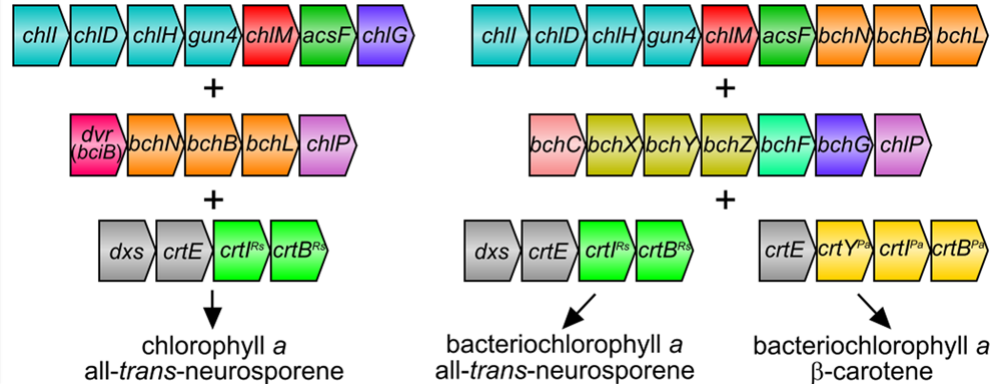

The biosynthesis of chlorophylls (Chls) and bacteriochlorophylls (BChls) represents a key aspect of photosynthesis research. Our previous work assembled the complete pathway for the synthesis of Chl *a* in *Escherichia coli*; here we engineer the more complex BChl *a* pathway in the same heterotrophic host. Coexpression of 18 genes enabled *E. coli* to produce BChl *a*, verifying that we have identified the minimum set of genes for the BChl *a* biosynthesis pathway. The protochlorophyllide reduction step was mediated by the *bchNBL* genes, and this same module was used to modify the Chl *a* pathway previously constructed in *E. coli*, eliminating the need for the light-dependent protochlorophyllide reductase. Furthermore, we demonstrate the feasibility of synthesizing more than one family of photosynthetic pigments in one host by engineering *E. coli* strains that accumulate the carotenoids neurosporene and β-carotene in addition to BChl *a*.

## Introduction

**Figure 1:**
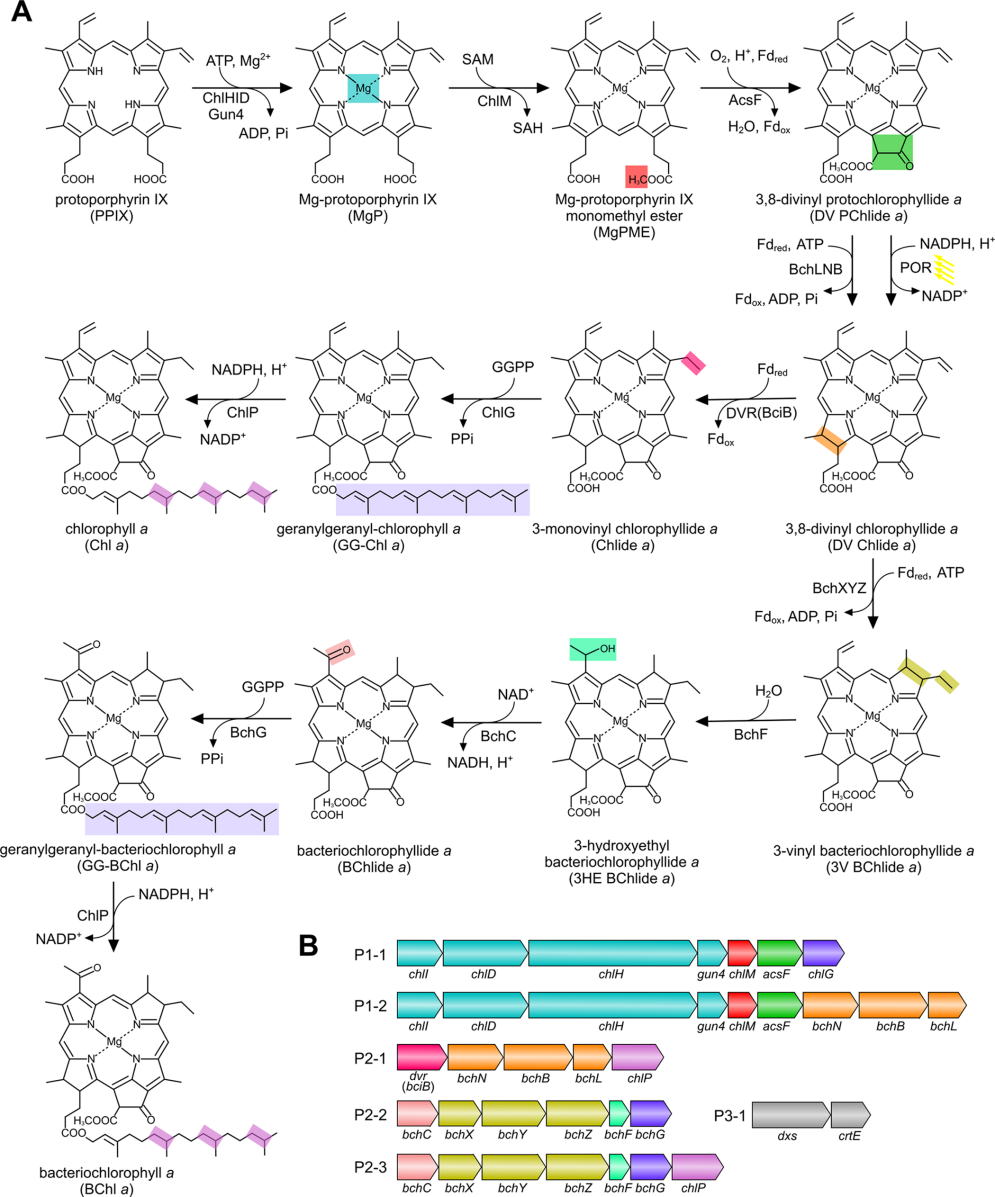
**(Bacterio)chlorophyll biosynthetic pathways assembled in *E. coli*.** (A) Overall reactions from PPIX to Chl *a* and BChl *a* catalyzed by the enzymes introduced to *E. coli*, with chemical change(s) of each step denotated by colored shading. ATP, adenosine triphosphate; ADP, adenosine diphosphate; Pi, inorganic phosphate; SAM, *S*-adenosine-l-methionine; SAH, *S*-adenosyl-l-homocysteine; Fd_red_, reduced ferredoxin; Fd_ox_, oxidized ferredoxin; GGPP, geranylgeranyl pyrophosphate; PPi, inorganic pyrophosphate; NADP^+^, nicotinamide adenine dinucleotide phosphate; NADPH, reduced form of NADP^+^; NAD^+^, nicotinamide adenine dinucleotide; NADH, reduced form of NAD^+^. (B) Arrangement and relative size of each gene in the constructed plasmids with the same color coding as in (A). P1-1 and P1-2 are pET3a-based plasmids created by the link and lock method with expression of all genes driven by a single T7 promoter and a ribosome binding site upstream of each gene. P2-1, P2-2, and P2-3 are based on a modified pCDFDuet1 vector for the link and lock cloning of multiple genes (*dvr* and *bchNBL* genes in P2-1 and *bchCXYZFG* genes in P2-2 and P2-3) at multiple cloning site (MCS) 1 with the T7*lac* promoter 1 replaced with a T7 promoter (see Methods for details). The *SynechocystischlP* gene was cloned at MCS2 in P2-1 and P2-3. P3-1 is a pCOLADuet1-based plasmid and contains the *E. colidxs* gene at MCS1 and the *Rvi. gelatinosuscrtE* gene at MCS2.

Photosynthesis is arguably the most important biochemical process on Earth, supplying the oxygen and organic compounds required for most life on Earth. In chlorophyll (Chl)-based photosynthesis, these pigments are often housed in various photosynthetic pigment–protein complexes to harvest solar energy and perform photochemistry. Due to its global significance, Chl biosynthesis has been extensively studied, leading to the identification and biochemical characterization of most of the biosynthetic enzymes, augmented in some cases by structural analysis.^1,2^ The tetrapyrrole molecule protoporphyrin IX (PPIX) (Figure 1A) is synthesized natively by *Escherichia coli* as the biosynthetic precursor of heme. It was shown that PPIX could also form the basis for the heterologous assembly of the entire biosynthetic pathway leading to Chl *a*.^3^ That work created a branchpoint in tetrapyrrole metabolism in *E. coli*, in which PPIX could form either heme or Chl *a*, and in doing so, it delineated a minimum set of enzymes required for Chl biosynthesis. PPIX is the biosynthetic precursor for both Chl and bacteriochlorophyll (BChl) biosynthesis, and the first step for both pathways is catalyzed by the magnesium chelatase complex. The multisubunit enzyme for the Chl pathway comprises ChlIDH and Gun4^4−8^ and inserts Mg^2+^ into the macrocyclic ring of PPIX to form the first Chl-specific intermediate, Mg-PPIX (MgP). MgP is then methylated at the C13 propionate side chain by MgP methyltransferase (ChlM).^9^ Oxidation and cyclization of the methylated C13 propionate, catalyzed by the MgP monomethyl ester (MgPME) cyclase (AcsF),^3,10,11^ produces the first green biosynthetic intermediate, 3,8-divinyl protochlorophyllide *a* (DV PChlide *a*). The C17=C18 double bond of DV PChlide *a* is reduced by the light-dependent protochlorophyllide oxidoreductase (LPOR) to generate 3,8-divinyl chlorophyllide *a* (DV Chlide *a*).^12−14^ Alternatively, the dark-operative POR (DPOR) converts DV PChlide *a* to DV Chlide *a* in a light-independent manner.^15^ The 8-vinyl group of DV Chlide *a* is then converted to an ethyl group by divinyl reductase (DVR) to form chlorophyllide *a* (Chlide *a*).^16,17^ Chlide *a* is the common precursor for the two most prominent Chl types, Chl *a* in oxygenic photosynthesis and BChl *a* in anoxygenic photosynthesis.^2^

Chl *a* and BChl *a* differ slightly in their macrocyclic structures, and additional modifications are required to convert Chlide *a* to bacteriochlorophyllide *a* (BChlide *a*). The C7=C8 double bond of Chlide *a* is reduced by the nitrogenase-like enzyme chlorophyllide *a* oxidoreductase (COR, BchXYZ), producing 3-vinyl bacteriochlorophyllide *a* (3V BChlide *a*).^18,19^ COR also has latent DVR activity and thus is able to use DV Chlide *a* as substrate to form 3V BChlide *a*.^20,21^ The 3-vinyl group undergoes hydroxylation by 3V BChlide *a* hydratase (BchF) to form a hydroxyethyl (HE) group, which is oxidized by 3HE BChlide *a* dehydrogenase (BchC) to form an acetyl group,^22^ thus producing BChlide *a*. Alternative reaction sequences are possible in which the COR step is preceded by the formation of the 3-HE or 3-acetyl from the 3-vinyl group.^22,23^ Chl *a* and BChl *a* share the final biosynthetic steps for attachment of a phytol moiety to the macrocycle^24^ (Figure 1A). In the Chl *a* branch, Chlide *a* is esterified with geranylgeranyl pyrophosphate (GGPP) by Chl synthase (ChlG). The GG tail is converted to a phytol group by three successive reductions by GG reductase (ChlP) to complete the biosynthesis of Chl *a*. The final steps of BChl *a* biosynthesis resemble those of Chl *a* with the two enzymes involved, BChl synthase (BchG) and GG reductase (BchP), being homologous to their respective ChlG and ChlP counterparts in Chl *a* biosynthesis. However, (B)Chl synthases have a high degree of specificity for their tetrapyrrole substrates; BChl synthase only utilizes BChlide *a*, and Chl synthase only utilizes Chlide *a*.^25^ So far, our understanding of the BChl *a* biosynthetic pathway has remained at the level of individual steps, and assembly of the complete BChl biosynthetic pathway in a heterologous organism has not been reported.

Apart from (B)Chls, photosynthesis also requires carotenoids for light harvesting, photoprotection, and stabilization of pigment–protein complexes.^26−29^ Carotenoids are isoprenoid compounds and absorb in the 400–550 nm region of the solar spectrum to supplement the spectral coverage of (B)Chls for photosynthesis. Carotenoids have a very wide structural diversity, with more than 1100 carotenoids found in nature to date. Because of their great commercial importance as nutritional supplements and food colorants, many carotenoids have been produced heterologously in engineered microbial hosts such as *E. coli* with high efficiencies as a result of extensive metabolic engineering.^30,31^ Elucidation of the biosynthetic pathway of a photosynthetic pigment is fundamental in photosynthesis research, as it not only provides the origin of the structural features of a pigment, which determines its function in photosynthesis, but also is a prerequisite for engineering the pathway in a heterologous host. Here we have assembled the biosynthetic pathway for Chl *a* and BChl *a* either on their own or in combination with the biosynthetic pathway for neurosporene (in the case of Chl *a* and BChl *a*) and β-carotene (in the case of BChl *a*) in *E. coli*. This work provides the basis for converting a heterotrophic host to a platform for studying the biosynthesis of photosynthetic pigments and assembly of pigment–protein complexes utilized by photosynthesis.

## Results and Discussion

### Engineering a Light-Independent Chl Biosynthetic Pathway in *E. coli*

We previously assembled a functional Chl biosynthetic pathway in *E. coli* with the genes sourced from the model cyanobacterium *Synechocystis* sp. PCC 6803 (hereafter *Synechocystis*) and the purple phototrophic bacterium *Rubrivivax gelatinosus*.^3^ It was necessary to expose the engineered cells to light in order to activate the light-dependent POR, which supplies Chlide for the heterologous synthesis of Chl *a*. Considering the phototoxic nature of Chl biosynthetic intermediates, a light-independent Chl biosynthetic pathway might be a better alternative (Figure 1A), which would require the enzyme DPOR. DPOR is a three-subunit enzyme, and activity has been demonstrated with subunits recombinantly produced in *E. coli*.^32^ However, it is unknown whether DPOR can exhibit *in vivo* activity when expressed in *E. coli* together with a number of other Chl biosynthetic enzymes.

**Figure 2:**
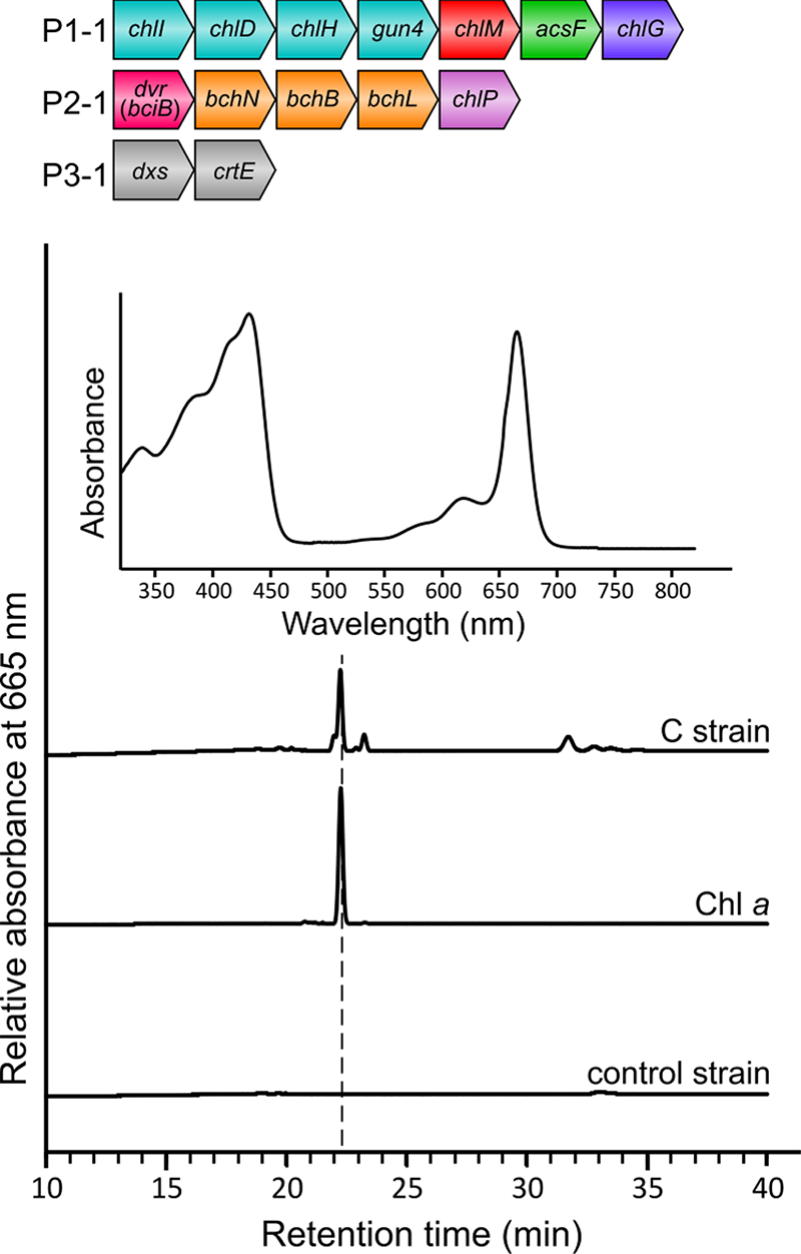
**HPLC analysis of pigments extracted from *E. coli* strains expressing chlorophyll biosynthetic genes.** Pigments were extracted from the C strain, which contained the P1-1, P2-1, and P3-1 plasmids. The *E. coli* control strain contained the empty pET3a, pCDFDuet1, and pCOLADuet1 vectors. Retention times and (inset) the absorbance spectra of the peaks were used to identify Chl *a*. Note that the 22.3 min peak in the elution profile of the C strain has an absorption spectrum identical to that of the Chl *a* standard isolated from *Synechocystis*.

To test the possibility of a DPOR-based Chl biosynthetic pathway, we modified the plasmid-based genetic module for Chl biosynthesis in *E. coli* to increase the number of genes that can be included (see Methods for details). The P1-1 plasmid was made by cloning the *SynechocytischlG* gene (see Table S1 for a list of genes used in this study) into the pET3a-based IA plasmid containing the *SynechocystischlI*, *chlD*, *chlH*, *gun4*, and *chlM* genes and the *Rvi. gelatinosusacsF* gene (Figure 1B). The P2-1 plasmid is based on a modified pCDFDuet1 vector with the *Synechocystisdvr* gene and the *bchN*, *bchB*, and *bchL* genes encoding DPOR from the purple phototrophic bacterium *Rhodobacter sphaeroides*, cloned at multiple cloning site (MCS) 1 by the link and lock method,^33^ and the *SynechocystischlP* gene cloned at MCS2. The pCOLADuet1-based DE plasmid, which contains the *E. colidxs* and *Rvi. gelatinosuscrtE* genes, was used previously to ensure a sufficient supply of GGPP, a substrate for Chl synthase.^3^ This plasmid was used in this study without any alteration but was renamed as the P3-1 plasmid for consistency. We conducted three sequential transformations to get the P1-1, P2-1, and P3-1 plasmids into the *E. coli* C43(DE3) strain.^34^ The final transformant, named the C strain, was assayed for its capacity to produce Chl *a* following the induction of the plasmid-borne genes with isopropyl-β-d-thiogalactopyranoside (IPTG). Pigments extracted from the harvested *E. coli* cells were analyzed by high-performance liquid chromatography (HPLC), with the elution profile monitored by the absorbance at 665 nm. No peak was detected for the control strain (Figure 2), which contained empty pET3a, pCDFDuet1, and pCOLADuet1 vectors. It is clear that the C strain accumulated Chl *a*, as shown by the 22.3 min peak, which has an elution time and absorption spectrum identical to those of the Chl *a* standard isolated from *Synechocystis* wild-type (WT) cells (Figure 2). The C strain under the tested conditions accumulated 839 ± 118 Chl *a* molecules per cell.

### Construction of the BChl Biosynthetic Pathway in *E. coli*

This success with the 14-gene DPOR-based Chl biosynthetic pathway demonstrated the potential of *E. coli* as a platform for building heterologous pathways with great complexity. Next, we went further to test the possibility of establishing the pathway for BChl *a*, the pivotal pigment utilized by anoxygenic phototrophs for light harvesting and photochemistry. The biosynthesis of BChl *a* shares the earlier steps with Chl *a* up to intermediate Chlide *a* but is considerably more complex; three extra steps are required to generate BChlide *a* by reducing the C7=C8 double bond and converting the 3-vinyl group to an acetyl (Figure 1A). In addition, the BChl synthase BchG and Chl synthase ChlG are homologous but differ in substrate specificity, so they are not interchangeable.^25^

**Figure 3:**
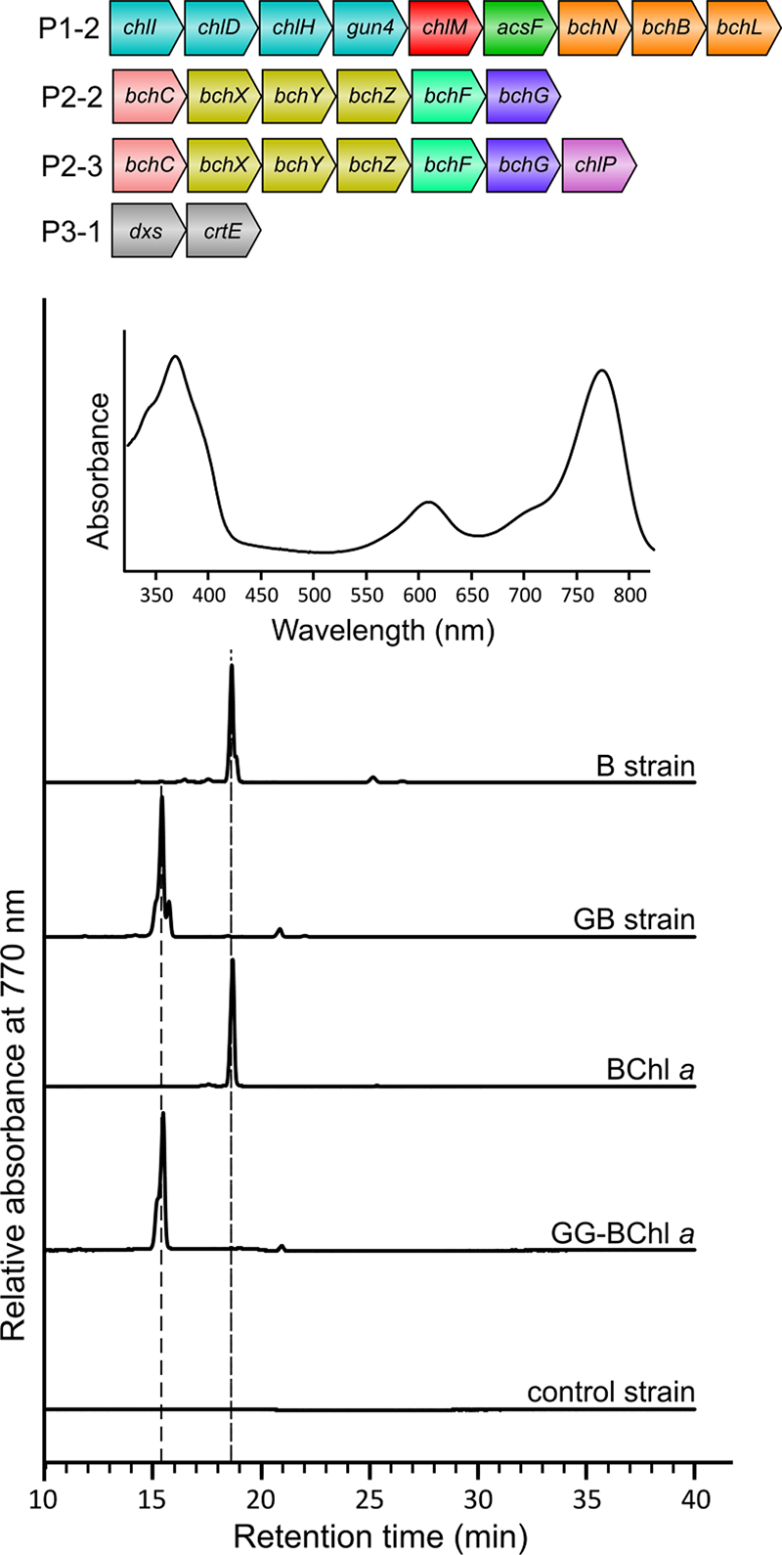
**HPLC analysis of pigments extracted from *E. coli* strains expressing bacteriochlorophyll biosynthetic genes.** Pigments were extracted from the GB strain containing the P1-2, P2-2, and P3-1 plasmids and the B strain containing the P1-2, P2-3, and P3-1 plasmids. The *E. coli* control strain contained the empty pET3a, pCDFDuet1, and pCOLADuet1 vectors. Retention times and (inset) the absorbance spectrum of the peaks were used to identify GG-BChl *a* and BChl *a*. Note that GG-BChl *a* and BChl *a* have the same absorbance spectrum. The 15.5 min peak in the elution profile of the GB strain and the 18.7 min peak for the B strain have absorbance spectra identical to those of the GG-BChl *a* and BChl *a* standards isolated from *Rba. sphaeroides* strains.

We cloned the *Rba. sphaeroidesbchN*, *bchB*, and *bchL* genes into the IA plasmid to obtain the P1-2 plasmid constituting a genetic module for DV Chlide biosynthesis (Figure 1B). The *Rba. sphaeroidesbchC*, *bchX*, *bchY*, *bchZ*, *bchF*, and *bchG* genes, encoding enzymes specific for BChl biosynthesis, were cloned into the modified pCDFDuet1 vector to form the P2-2 plasmid. The *SynechocystischlP* gene was cloned at MCS2 of P2-2 to produce the P2-3 plasmid. We sequentially transformed the P1-2, P2-2, and P3-1 plasmids into *E. coli* C43(DE3) to generate the GB strain. *In vivo* pigment production assays and subsequent HPLC analysis revealed that the GB strain accumulated a pigment species eluting at 15.5 min that absorbs maximally at 365 and 770 nm, corresponding to the Soret and Q_*y*_ bands of BChlide *a*, respectively (Figure 3). We further identified the pigment species as GG-BChl *a* using a pigment standard isolated from a Δ*bchP* mutant^35^ of *Rba. sphaeroides*. The GB strain synthesized 14056 ± 1843 GG-BChl *a* molecules per cell under the tested conditions. Replacement of the P2-2 plasmid in the GB strain with P2-3 resulted in the B strain. The detection of BChl *a* (pigment standard isolated from *Rba. sphaeroides* WT cells) in this strain, which eluted at 18.7 min (Figure 3), demonstrates that a complete BChl biosynthetic pathway has been successfully assembled in *E. coli*. The B strain produced 3204 ± 463 BChl *a* molecules per cell.

### Combination of the (B)Chl and Carotenoid Biosynthetic Pathways in *E. coli*

**Figure 4:**
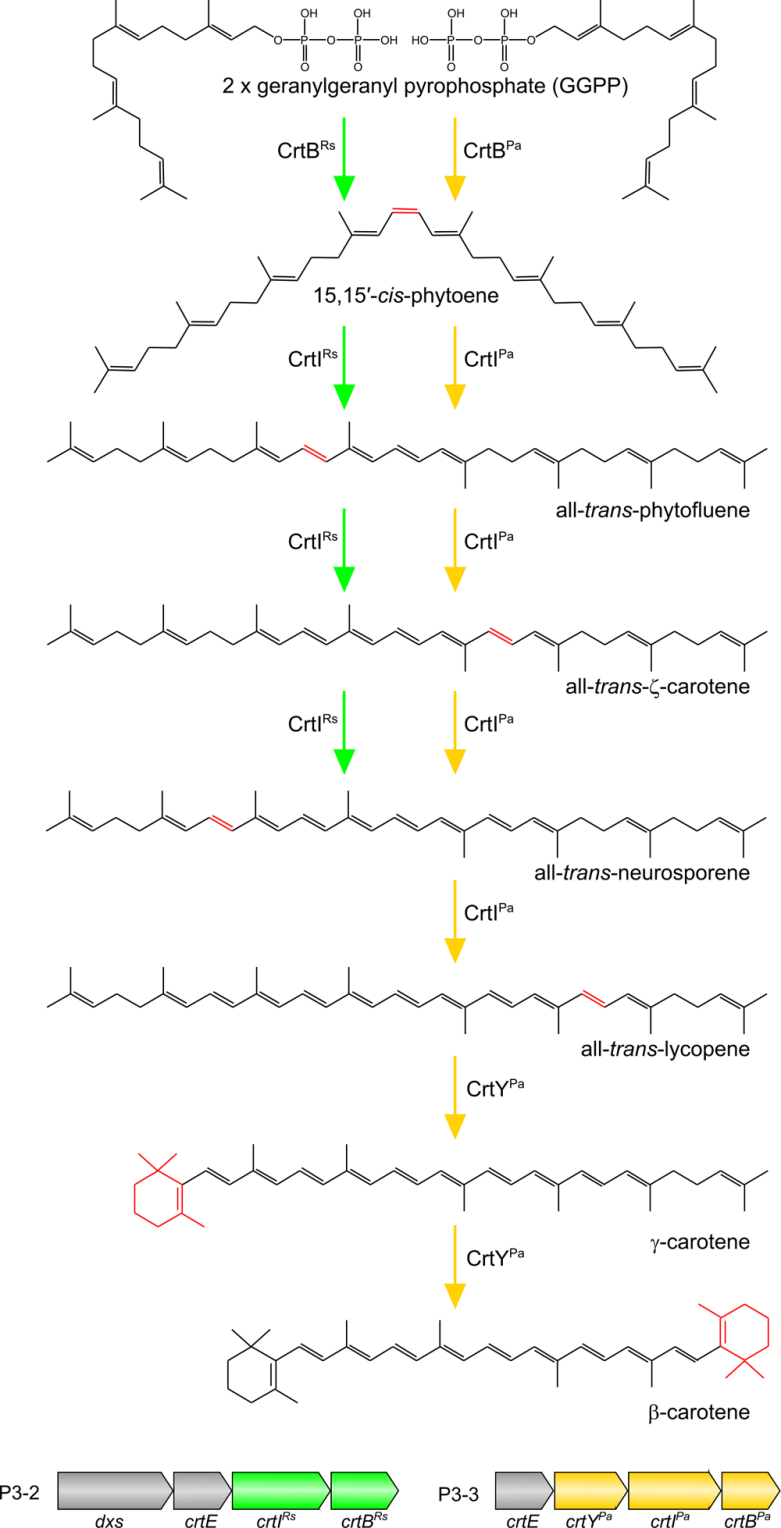
**Conversion of geranylgeranyl pyrophosphate to neurosporene and β-carotene catalyzed by the enzymes introduced to *E. coli***. Green arrows indicate the route to neurosporene with the *Rba. sphaeroides* carotenoid biosynthetic enzymes including CrtI^Rs^, a three-step phytoene desaturase. Orange arrows indicate the β-carotene biosynthetic pathway with the enzymes sourced from *Pantoea agglomerans*, including a four-step phytoene desaturase (CrtI^Pa^). Chemical change(s) of each step are indicated in red. P3-2 is based on the pCOLADuet1 vector with the *E. colidxs* gene at MCS1 and the *Rvi. gelatinosuscrtE* gene and the *Rba. sphaeroidescrtIB* genes (as a single fragment fused by overlap extension PCR) at MCS2. P3-3 is based on a modified pCOLADuet1 vector for link and lock cloning of the *P. agglomeranscrtYIB* genes (as a single fragment) downstream of the *Rvi. gelatinosuscrtE* gene at MCS1 (see Methods for details).

**Figure 5:**
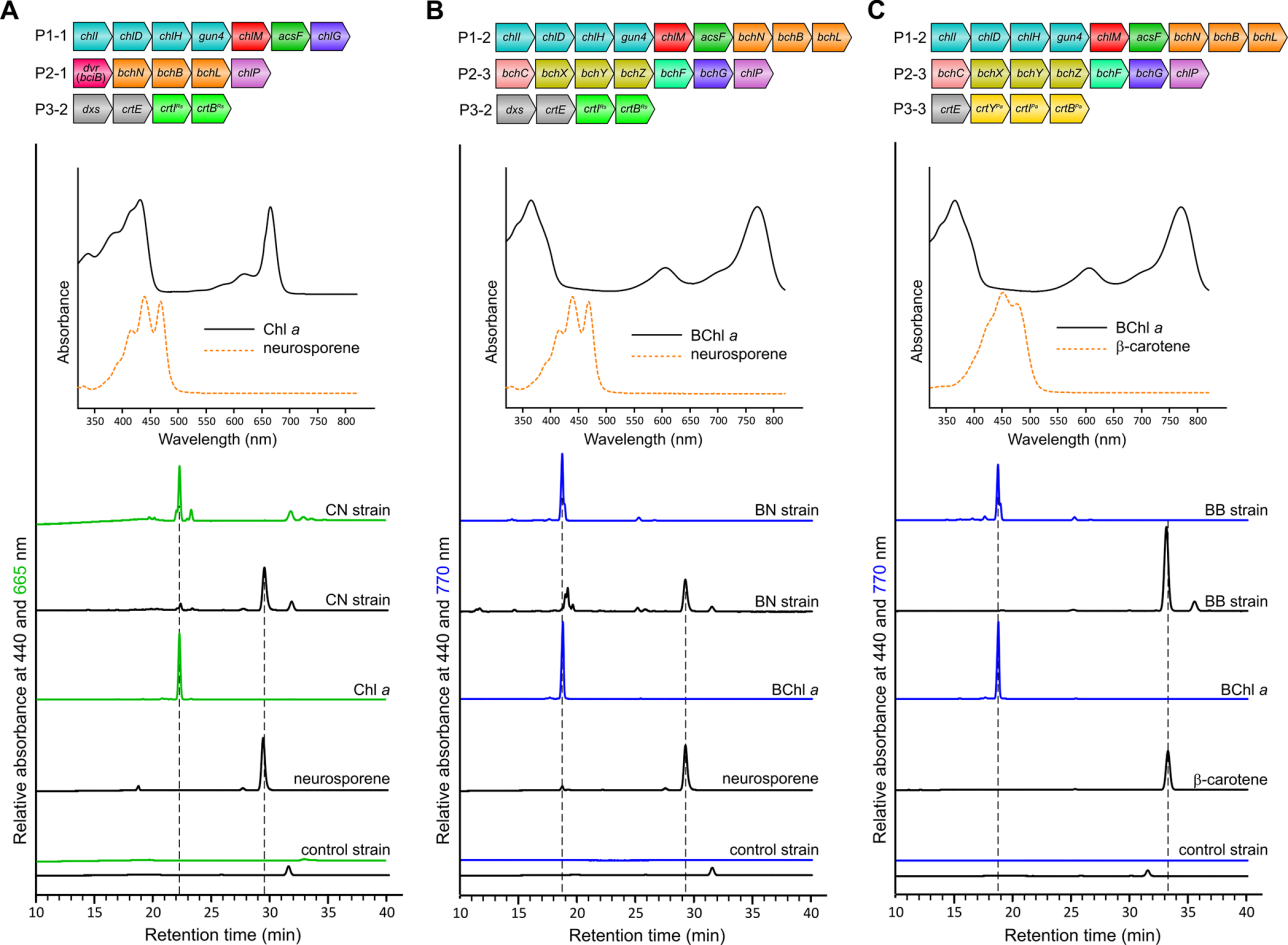
**HPLC analysis of pigment production of *E. coli* strains with introduced (bacterio)chlorophyll and carotenoid biosynthetic pathways.** Elution of pigments was monitored by absorbance at 440 nm (shown in black), 665 nm (shown in green), and 770 nm (shown in blue). The *E. coli* control strain contained the empty pET3a, pCDFDuet1, and pCOLADuet1 vectors. Pigment species were identified by comparing their retention times and (insets) absorbance spectra with those of the pigment standards. (A) Accumulation of Chl *a* and neurosporene in the CN strain containing the P1-1, P2-1, and P3-2 plasmids. (B) Accumulation of BChl *a* and neurosporene in the BN strain containing the P1-2, P2-3, and P3-2 plasmids. (C) Accumulation of BChl *a* and β-carotene in the BB strain containing the P1-2, P2-3, and P3-3 plasmids.

After engineering the (B)Chl biosynthetic pathways in *E. coli*, we went on to test whether (B)Chl can be cosynthesized with carotenoids in a heterotrophic host, which would mimic natural photosynthetic organisms. We began with the carotenoid neurosporene for the simplicity of its biosynthetic pathway; only two enzymes, the phytoene synthase (CrtB) and the three-step phytoene desaturase (CrtI), are required to form neurosporene from the major carotenoid precursor GGPP (Figure 4). We replaced the *crtE* gene in the P3-1 plasmid with a single gene fragment containing the *Rvi. gelatinosuscrtE* gene and the *Rba. sphaeroidescrtIB* genes to get the P3-2 plasmid (Figure 4; see Methods for details). We transformed the P1-1, P2-1, and P3-2 plasmids into *E. coli* C43(DE3) to generate the CN strain. Using pigment standards isolated from photosynthetic organisms with Chl *a* from *Synechocystis* WT cells and neurosporene from a Δ*crtC* mutant^36^ of *Rba. sphaeroides*, we confirmed that the CN strain produced Chl *a*, as shown by the 22.3 min peak in the 665 nm elution profile, and neurosporene, as shown by the 29.2 min peak in the 440 nm elution profile (Figure 5A). The yields of Chl *a* and neurosporene for the CN strain were determined to be 415 ± 107 and 3337 ± 1134 molecules per cell, respectively. Furthermore, neurosporene can also be produced together with BChl *a* in *E. coli*, as demonstrated by the pigment profile of the BN strain, which contained the P1-2, P2-3, and P3-2 plasmids (Figure 5B). The BN strain synthesized 8887 ± 1074 BChl *a* and 6547 ± 407 neurosporene molecules per cell. The BN strain produced 2.8 times as much BChl *a* as the B strain, indicating that the inclusion of the neurosporene biosynthesis pathway may boost the production of BChl *a*; the basis for this effect requires future investigation.

We then chose β-carotene, a ubiquitous and important type of carotenoid found in photosynthetic organisms. The biosynthesis of β-carotene requires the four-step phytoene desaturase (instead of the three-step enzyme involved in neurosporene biosynthesis) as well as the lycopene β-cyclase (CrtY) to form the β-ring at both ends in the structure (Figure 4). We amplified the genes encoding these enzymes from the reported pAC-BETA plasmid,^37^ which contains the β-carotene biosynthetic genes from the carotenogenic non-photosynthetic bacterium *Pantoea agglomerans*. The resulting *crtYIB* gene fragment was then cloned with the *Rvi. gelatinosuscrtE* gene into a modified pCOLADuet1 vector using the link and lock method to get the P3-3 plasmid (Figure 4). The P3-3 plasmid was combined with the P1-2 and P2-3 plasmids to produce the BB strain. HPLC analysis of pigments extracted from this strain revealed BChl *a*, as indicated by an 18.7 min peak in the 770 nm elution profile, and a 33.2 min peak in the 440 nm elution profile (Figure 5C), which was identified as β-carotene using a pigment standard (Sigma-Aldrich C4582). The pigment level of the BB strain was quantified as 2773 ± 154 BChl *a* and 82795 ± 5166 β-carotene molecules per cell. The BChl *a* level of the BB strain is only slightly lower than that of the B strain (3204 ± 463 molecules per cell).

## Concluding Remarks

To summarize, this study reports the assembly of the entire biosynthetic pathway for BChl *a* in a non-photosynthetic organism, which not only validates our knowledge regarding the biosynthesis of BChl *a* but also opens up the possibility of assembling BChl *a*-based pigment–protein complexes in heterotrophic organisms. We also modified the previous *E. coli* light-dependent Chl *a* pathway to allow it to function in the dark. In addition, we have combined each of the (B)Chl biosynthetic pathways, the “Chl *a* module” or the “BChl *a* module”, with one of two carotenoid biosynthetic pathways, the “neurosporene module” and “β-carotene module”, to create *E. coli* strains that can synthesize (B)Chl and carotenoids simultaneously. This represents a proof of principle for integrating various cofactor biosynthetic modules into a heterotrophic platform for photosystem assembly. Compared to the native host, *Rba. sphaeroides*, which has a BChl content of 2.1 million molecules per cell^38^ and a similar carotenoid content, the pigment productivity of our *E. coli* strains is low. It is worth noting that the *Rba. sphaeroides* mutants with the genes encoding subunits of the photosynthetic apparatus deleted have much lower pigment levels than the WT. Thus, to increase the BChl and carotenoid yields in *E. coli*, future efforts should be made not only in the optimization of pigment biosynthetic pathways but also in the introduction of pigment-binding proteins.

## Methods

### Bacterial Strains and Plasmids

Bacterial strains and plasmids used in this study are listed in Table S2. Primers used for plasmid construction are listed in Table S3. *E. coli* strains were grown at 37 °C in LB medium when generating plasmid constructs or in a modified Terrific Broth medium with glycerol added at 0.8% v/v for *in vivo* assays of pigment production. If required, antibiotics were added at 30 μg mL^–1^ for kanamycin, 100 μg mL^–1^ for ampicillin, and 25 μg mL^–1^ for streptomycin. *Rba. sphaeroides* strains were grown in M22+ medium supplemented with 0.1% w/v casamino acids at 30 °C as described previously.^39^*Synechocystis* strains were grown in BG-11 medium^40^ buffered with 10 mM TES (pH 8.2, adjusted by KOH) at 30 °C with continuous illumination. Glucose was added at a concentration of 5 mM if required.

The *SynechocystischlG* gene was excised from the pET3a-*chlG* plasmid^3^ with *Xba*I/*Hind*III and cloned into the *Spe*I/*Hind*III-digested IA plasmid^3^ by the link and lock method,^33^ resulting the P1-1 plasmid. The *bchNB* genes were amplified as a single fragment from *Rba. sphaeroides* genomic DNA with the internal *Nde*I and *Hind*III sites removed by site-directed mutagenesis to facilitate subsequent cloning. The *bchL* gene with its 61 bp upstream sequence was amplified from *Rba. sphaeroides* genomic DNA. The resulting *bchNB* and *bchL* gene fragments were fused by overlap extension PCR, digested with *Nde*I/*Spe*I, and cloned into *Nde*I/*Spe*I-digested pET3a to construct pET3a-*bchNBL*. Then the *bchNBL* gene fragment was cloned into the IA plasmid as generating P1-1 to get the P1-2 plasmid.

The *Xba*I–*Hind*III region of pCDFDuet1 containing the *lacI* gene and T7*lac* promoter 1 was replaced with the T7 promoter–*Xba*I–*Hind*III region of pET3a-*dvr*^3^ to generate pCDFDuet1*-*dvr.* The *SynechocystischlP* gene was excised from the BoP plasmid^3^ and cloned into the *Hind*III/*Xho*I sites of pCDFDuet1*-*dvr* to form pCDFDuet1*-*dvr*-2-*chlP*. The *bchNBL* gene fragment was cloned downstream of the *dvr* gene of pCDFDuet1*-*dvr*-2-*chlP* by the link and lock method to form the P2-1 plasmid. The *bchCXYZ* genes were amplified as a single fragment from *Rba. sphaeroides* genomic DNA, digested with *Nde*I/*Spe*I, and cloned into *Nde*I/*Spe*I-digested pET3a to generate pET3a-*bchCXYZ*. The *bchF* and *bchG* genes were amplified from *Rba. sphaeroides* genomic DNA, digested with *Nde*I/*Spe*I, and cloned into *Nde*I/*Spe*I-digested pET3a to get pET3a-*bchF* and pET3a-*bchG*, respectively. The *bchF* and *bchG* genes were cut from the pET3a constructs and adjoined one by one using the link and lock method to construct pET3a-*bchCXYZFG*. The *bchCXYZFG* fragment was cut from pET3a-*bchCXYZFG* by *Xba*I/*Hind*III and used to replace the *dvr* gene in pCDFDuet1*-*dvr* and pCDFDuet1*-*dvr*-2*-chlP* to construct the P2-2 plasmid and P2-3 plasmids, respectively.

The DE plasmid reported previously^3^ is renamed P3-1 in this study for consistency. The *E. colidxs* gene was subcloned from P3-1 into the *Nco*I/*Hind*III sites of pCOLADuet1 to form pCOLADuet1-*dxs*. The *Rvi. gelatinosuscrtE* gene, amplified from P3-1, and the *crtIB* genes, amplified as a single fragment from *Rba. sphaeroides* genomic DNA, were fused by overlap extension PCR with a 42 bp ribosome binding site sequence (with the last T changed to A) from pET3a placed between the *crtE* and *crtI* genes. The resulting *crtEIB* gene fragment was cloned into the *Nde*I/*Kpn*I sites of pCOLADuet1-*dxs* to obtain the P3-2 plasmid. The *Rvi. gelatinosuscrtE* gene was amplified from P3-1, digested with *Nde*I/*Spe*I, and cloned into *Nde*I/*Spe*I-digested pET3a to make pET3a-*crtE*. The *crtE* gene with the adjacent *Spe*I–*Hind*III region of pET3a was amplified from pET3a-*crtE* and cloned into the *Nco*I/*Hind*III sites of pCOLADuet1 to form pCOLADuet1*-*crtE*. The *P. agglomeranscrtYIB* genes were amplified from pAC-BETA^37^ as a single fragment, digested with *Nde*I/*Spe*I, and cloned into *Nde*I/*Spe*I-digested pET3a to get pET3a-*crtYIB*. The *crtYIB* gene fragment was cloned downstream of the *crtE* gene in pCOLADuet1*-*crtE* by the link and lock method to obtain the P3-3 plasmid.

### *In Vivo* Assays for Pigment Production in *E. coli*

*E. coli* C43(DE3) was sequentially transformed with the pET3a-, pCDFDuet1-, and pCOLADuet1-based plasmids and selected on LB agar with appropriate antibiotics. *E. coli* strains harboring three plasmids were assayed for pigment production. For *in vivo* assays, a single colony was used to inoculate 10 mL of LB medium with 100 μg mL^–1^ ampicillin, 25 μg mL^–1^ streptomycin, and 30 μg mL^–1^ kanamycin and grown overnight at 37 °C with shaking at 220 rpm. The next day, 100 μL of the resulting culture was used to inoculate 10 mL of TB medium with antibiotics in 50 mL Falcon tubes and grown as above for 4 h. Then the culture was cooled at room temperature for 15 min before induction with 0.5 mM IPTG. ALA and Mg^2+^ (equimolar mixture of MgCl_2_ and MgSO_4_) were also added at 10 mM at the point of induction, and the culture was incubated for a further 24 h at 30 °C in the dark, with shaking at 175 rpm, before pigments were extracted from the cells.

### Pigment Extraction and Analysis by HPLC

*E. coli* cells were harvested from liquid cultures and washed once in 25 mM HEPES-NaOH buffer (pH 7.4). Pigments were extracted with an excess of 7:2 v/v acetone/methanol by vigorous shaking using a tissue grinder (Tiss-32, Jingxin, Shanghai), incubated on ice for 10 min, and centrifuged at 16,000 *g* for 5 min at 4 °C. The resulting supernatant containing extracted pigments was transferred to a new tube and analyzed immediately or vacuum-dried using a vacuum centrifuge (MC-2, Jiaimu, Beijing) and stored at −20 °C for future analysis. Chl *a*, BChl *a*, GG-BChl *a*, and neurosporene were extracted from *Synechocystis* WT, *Rba. sphaeroides* WT, a *Rba. sphaeroides* Δ*bchP* mutant,^35^ and a *Rba. sphaeroides* Δ*crtC* mutant,^36^ respectively. The β-carotene pigment standard was purchased from Sigma-Aldrich (C4582). A pigment solution in 7:2 v/v acetone/methanol, either freshly extracted or reconstituted from dried sample, was analyzed on an Agilent 1260 HPLC system equipped with a diode array detector. A published method^41^ with some modifications was used for separation of (B)Chl *a* and carotenoids. The pigment solution was loaded onto a Phenomenex Luna C18(2) reversed-phase column (5 μm particle size, 100 Å pore size, 250 mm × 4.6 mm). Solvent A was 64:16:20 v/v/v methanol/acetone/H_2_O, and solvent B was 80:20 v/v methanol/acetone. Pigment species were eluted at 40 °C at a flow rate of 1 mL min^–1^ with a linear gradient of 50–100% solvent B over 14 min, followed by further elution with 100% solvent B for 23 min. The eluates were monitored by absorbance at 440, 665, and 770 nm.

## Supporting Information Available

Genes used to assemble (B)Chl and carotenoid biosynthetic pathways in *E. coli* (Table S1); strains and plasmids described in this study (Table S2); oligonucleotide primers used in this study (Table S3) (PDF)

## References

[ref1] Fujita Y.; Yamakawa H. Biochemistry of chlorophyll biosynthesis in photosynthetic prokaryotes. In Modern Topics in the Phototrophic Prokaryotes: Metabolism, Bioenergetics, and Omics; Hallenbeck P., Ed.; Springer: Cham, Switzerland, 2017; pp 67–122.

[ref2] Bryant D. A.; Hunter C. N.; Warren M. J. Biosynthesis of the modified tetrapyrroles—the pigments of life. J. Biol. Chem. 2020, 295, 6888–6925. 10.1074/jbc.REV120.006194. 32241908 PMC7242693

[ref3] Chen G. E.; Canniffe D. P.; Barnett S. F. H.; Hollingshead S.; Brindley A. A.; Vasilev C.; Bryant D. A.; Hunter C. N. Complete enzyme set for chlorophyll biosynthesis in *Escherichia coli*. Sci. Adv. 2018, 4, eaaq1407 10.1126/sciadv.aaq1407. 29387799 PMC5787379

[ref4] Chen X.; Pu H.; Wang X.; Long W.; Lin R.; Liu L. Crystal structures of GUN4 in complex with porphyrins. Mol. Plant 2015, 8, 1125–1127. 10.1016/j.molp.2015.04.013. 25958236

[ref5] Chen X.; Pu H.; Fang Y.; Wang X.; Zhao S.; Lin Y.; Zhang M.; Dai H. E.; Gong W.; Liu L. Crystal structure of the catalytic subunit of magnesium chelatase. Nat. Plants 2015, 1, 15125 10.1038/nplants.2015.125. 27250678

[ref6] Farmer D. A.; Brindley A. A.; Hitchcock A.; Jackson P. J.; Johnson B.; Dickman M. J.; Hunter C. N.; Reid J. D.; Adams N. B. P. The ChlD subunit links the motor and porphyrin binding subunits of magnesium chelatase. Biochem. J. 2019, 476, 1875–1887. 10.1042/BCJ20190095. 31164400 PMC6604950

[ref7] Adams N. B. P.; Bisson C.; Brindley A. A.; Farmer D. A.; Davison P. A.; Reid J. D.; Hunter C. N. The active site of magnesium chelatase. Nat. Plants 2020, 6, 1491–1502. 10.1038/s41477-020-00806-9. 33257858

[ref8] Gao Y. S.; Wang Y. L.; Wang X.; Liu L. Hexameric structure of the ATPase motor subunit of magnesium chelatase in chlorophyll biosynthesis. Protein Sci. 2020, 29, 1026–1032. 10.1002/pro.3816. PMC7096714 31891428

[ref9] Chen X.; Wang X.; Feng J.; Chen Y.; Fang Y.; Zhao S.; Zhao A.; Zhang M.; Liu L. Structural insights into the catalytic mechanism of *Synechocystis* magnesium protoporphyrin IX *O*-methyltransferase (ChlM). J. Biol. Chem. 2014, 289, 25690–25698. 10.1074/jbc.M114.584920. 25077963 PMC4162172

[ref10] Chen G. E.; Canniffe D. P.; Hunter C. N. Three classes of oxygen-dependent cyclase involved in chlorophyll and bacteriochlorophyll biosynthesis. Proc. Natl. Acad. Sci. U. S. A. 2017, 114, 6280–6285. 10.1073/pnas.1701687114. 28559347 PMC5474816

[ref11] Chen G. E.; Adams N. B. P.; Jackson P. J.; Dickman M. J.; Hunter C. N. How the O_2_-dependent Mg-protoporphyrin monomethyl ester cyclase forms the fifth ring of chlorophylls. Nat. Plants 2021, 7, 365–375. 10.1038/s41477-021-00876-3. 33731920 PMC7610348

[ref12] Sytina O. A.; Heyes D. J.; Hunter C. N.; Alexandre M. T.; van Stokkum I. H. M.; van Grondelle R.; Groot M. L. Conformational changes in an ultrafast light-driven enzyme determine catalytic activity. Nature 2008, 456, 1001–1004. 10.1038/nature07354. 19092933

[ref13] Zhang S.; Heyes D. J.; Feng L.; Sun W.; Johannissen L. O.; Liu H.; Levy C. W.; Li X.; Yang J.; Yu X.; Lin M.; Hardman S. J. O.; Hoeven R.; Sakuma M.; Hay S.; Leys D.; Rao Z.; Zhou A.; Cheng Q.; Scrutton N. S. Structural basis for enzymatic photocatalysis in chlorophyll biosynthesis. Nature 2019, 574, 722–725. 10.1038/s41586-019-1685-2. 31645759

[ref14] Dong C. S.; Zhang W. L.; Wang Q.; Li Y. S.; Wang X.; Zhang M.; Liu L. Crystal structures of cyanobacterial light-dependent protochlorophyllide oxidoreductase. Proc. Natl. Acad. Sci. U. S. A. 2020, 117, 8455–8461. 10.1073/pnas.1920244117. 32234783 PMC7165480

[ref15] Muraki N.; Nomata J.; Ebata K.; Mizoguchi T.; Shiba T.; Tamiaki H.; Kurisu G.; Fujita Y. X-ray crystal structure of the light-independent protochlorophyllide reductase. Nature 2010, 465, 110–114. 10.1038/nature08950. 20400946

[ref16] Chew A. G. M.; Bryant D. A. Characterization of a plant-like protochlorophyllide *a* divinyl reductase in green sulfur bacteria. J. Biol. Chem. 2007, 282, 2967–2975. 10.1074/jbc.M609730200. 17148453

[ref17] Saunders A. H.; Golbeck J. H.; Bryant D. A. Characterization of BciB: a ferredoxin-dependent 8-vinyl-protochlorophyllide reductase from the green sulfur bacterium *Chloroherpeton thalassium*. Biochemistry 2013, 52, 8442–8451. 10.1021/bi401172b. 24151992

[ref18] Nomata J.; Mizoguchi T.; Tamiaki H.; Fujita Y. A second nitrogenase-like enzyme for bacteriochlorophyll biosynthesis: Reconstitution of chlorophyllide *a* reductase with purified X-protein (BchX) and YZ-protein (BchY-BchZ) from *Rhodobacter capsulatus*. J. Biol. Chem. 2006, 281, 15021–15028. 10.1074/jbc.M601750200. 16571720

[ref19] Kiesel S.; Watzlich D.; Lange C.; Reijerse E.; Brocker M. J.; Rudiger W.; Lubitz W.; Scheer H.; Moser J.; Jahn D. Iron-sulfur cluster-dependent catalysis of chlorophyllide *a* oxidoreductase from *Roseobacter denitrificans*. J. Biol. Chem. 2015, 290, 1141–1154. 10.1074/jbc.M114.617761. 25422320 PMC4294481

[ref20] Tsukatani Y.; Yamamoto H.; Harada J.; Yoshitomi T.; Nomata J.; Kasahara M.; Mizoguchi T.; Fujita Y.; Tamiaki H. An unexpectedly branched biosynthetic pathway for bacteriochlorophyll *b* capable of absorbing near-infrared light. Sci. Rep. 2013, 3, 1217 10.1038/srep01217. 23386973 PMC3564038

[ref21] Harada J.; Mizoguchi T.; Tsukatani Y.; Yokono M.; Tanaka A.; Tamiaki H. Chlorophyllide *a* oxidoreductase works as one of the divinyl reductases specifically involved in bacteriochlorophyll *a* biosynthesis. J. Biol. Chem. 2014, 289, 12716–12726. 10.1074/jbc.M113.546739. 24637023 PMC4007461

[ref22] Lange C.; Kiesel S.; Peters S.; Virus S.; Scheer H.; Jahn D.; Moser J. Broadened substrate specificity of 3-hydroxyethyl bacteriochlorophyllide *a* dehydrogenase (BchC) indicates a new route for the biosynthesis of bacteriochlorophyll. a. J. Biol. Chem. 2015, 290, 19697–19709. 10.1074/jbc.M115.660555. 26088139 PMC4528133

[ref23] Pudek M. R.; Richards W. R. A possible alternate pathway of bacteriochlorophyll biosynthesis in a mutant of *Rhodopseudomonas sphaeroides*. Biochemistry 1975, 14, 3132–3137. 10.1021/bi00685a015. 1080053

[ref24] Proctor M. S.; Sutherland G. A.; Canniffe D. P.; Hitchcock A. The terminal enzymes of (bacterio) chlorophyll biosynthesis. R. Soc. Open Sci. 2022, 9, 211903 10.1098/rsos.211903. 35573041 PMC9066304

[ref25] Kim E. J.; Lee J. K. Competitive inhibitions of the chlorophyll synthase of *Synechocystis* sp. strain PCC 6803 by bacteriochlorophyllide *a* and the bacteriochlorophyll synthase of *Rhodobacter sphaeroides* by chlorophyllide *a*. J. Bacteriol. 2010, 192, 198–207. 10.1128/JB.01271-09. 19880605 PMC2798255

[ref26] Siefermann-Harms D. The light-harvesting and protective functions of carotenoids in photosynthetic membranes. Physiol. Plant. 1987, 69, 561–568. 10.1111/j.1399-3054.1987.tb09240.x.

[ref27] Paulsen H. Carotenoids and the assembly of light-harvesting complexes. In The Photochemistry of Carotenoids; Frank H. A., Young A. J., Britton G., Cogdell R. J., Eds.; Springer: Dordrecht, The Netherlands, 1999; pp 123–135.

[ref28] Hashimoto H.; Uragami C.; Cogdell R. J. Carotenoids and photosynthesis. In Carotenoids in Nature; Stange C., Ed.;. Subcellular Biochemistry, Vol. 79; Springer: Cham, Switzerland, 2016; pp 111–139. 10.1007/978-3-319-39126-7_4 27485220

[ref29] Polívka T.; Frank H. A. Molecular factors controlling photosynthetic light harvesting by carotenoids. Acc. Chem. Res. 2010, 43, 1125–1134. 10.1021/ar100030m. 20446691 PMC2923278

[ref30] Wang C.; Zhao S.; Shao X.; Park J. B.; Jeong S. H.; Park H. J.; Kwak W. J.; Wei G.; Kim S. W. Challenges and tackles in metabolic engineering for microbial production of carotenoids. Microb. Cell Fact. 2019, 18, 55 10.1186/s12934-019-1105-1. 30885243 PMC6421696

[ref31] Li C.; Swofford C. A.; Sinskey A. J. Modular engineering for microbial production of carotenoids. Metab. Eng. Commun. 2020, 10, e00118 10.1016/j.mec.2019.e00118. 31908924 PMC6938962

[ref32] Yamamoto H.; Nomata J.; Fuita Y. Functional expression of nitrogenase-like protochlorophyllide reductase from *Rhodobacter capsulatus* in *Escherichia coli*. Photochem. Photobiol. Sci. 2008, 7, 1238–1242. 10.1039/b802427h. 18846289

[ref33] McGoldrick H. M.; Roessner C. A.; Raux E.; Lawrence A. D.; McLean K. J.; Munro A. W.; Santabarbara S.; Rigby S. E.; Heathcote P.; Scott A. I.; Warren M. J. Identification and characterization of a novel vitamin B_12_ (cobalamin) biosynthetic enzyme (CobZ) from *Rhodobacter capsulatus*, containing flavin, heme, and Fe-S cofactors. J. Biol. Chem. 2005, 280, 1086–1094. 10.1074/jbc.M411884200. 15525640

[ref34] Miroux B.; Walker J. E. Over-production of proteins in *Escherichia coli*: mutant hosts that allow synthesis of some membrane proteins and globular proteins at high levels. J. Mol. Biol. 1996, 260, 289–298. 10.1006/jmbi.1996.0399. 8757792

[ref35] Hitchcock A.; Jackson P. J.; Chidgey J. W.; Dickman M. J.; Hunter C. N.; Canniffe D. P. Biosynthesis of chlorophyll *a* in a purple bacterial phototroph and assembly into a plant chlorophyll-protein complex. ACS Synth. Biol. 5 2016, 5, 948–954. 10.1021/acssynbio.6b00069. 27171912

[ref36] Chi S. C.; Mothersole D. J.; Dilbeck P.; Niedzwiedzki D. M.; Zhang H.; Qian P.; Vasilev C.; Grayson K. J.; Jackson P. J.; Martin E. C.; Li Y.; Holten D.; Hunter C. N. Assembly of functional photosystem complexes in *Rhodobacter sphaeroides* incorporating carotenoids from the spirilloxanthin pathway. Biochim. Biophys. Acta 2015, 1847, 189–201. 10.1016/j.bbabio.2014.10.004. 25449968 PMC4331045

[ref37] Cunningham F. X.; Pogson B.; Sun Z.; McDonald K. A.; DellaPenna D.; Gantt E. Functional analysis of the β and ε lycopene cyclase enzymes of *Arabidopsis* reveals a mechanism for control of cyclic carotenoid formation. Plant Cell 1996, 8, 1613–1626. 10.1105/tpc.8.9.1613. 8837512 PMC161302

[ref38] Hunter C. N.; Kramer H. J. M.; van Grondelle R. Linear dichroism and fluorescence emission of antenna complexes during photosynthetic unit assembly in *Rhodopseudomonas sphaeroides*. Biochim. Biophys. Acta 1985, 807, 44–51. 10.1016/0005-2728(85)90051-9.

[ref39] Chen G. E.; Canniffe D. P.; Martin E. C.; Hunter C. N. Absence of the *cbb*_3_ terminal oxidase reveals an active oxygen-dependent cyclase involved in bacteriochlorophyll biosynthesis in *Rhodobacter sphaeroides*. J. Bacteriol. 2016, 198, 2056–2063. 10.1128/JB.00121-16. 27215788 PMC4944227

[ref40] Rippka R.; Deruelles J.; Waterbury J. B.; Herdman M.; Stanier R. Y. Generic assignments, strain histories and properties of pure cultures of cyanobacteria. J. Gen. Microbiol. 1979, 111, 1–61. 10.1099/00221287-111-1-1.

[ref41] Canniffe D. P.; Thweatt J. L.; Chew A. G. M.; Hunter C. N.; Bryant D. A. A paralog of a bacteriochlorophyll biosynthesis enzyme catalyzes the formation of 1,2-dihydrocarotenoids in green sulfur bacteria. J. Biol. Chem. 2018, 293, 15233–15242. 10.1074/jbc.RA118.004672. 30126840 PMC6166724

