# Supplementary material for: Engineering Chlorophyll, Bacteriochlorophyll, and Carotenoid Biosynthetic Pathways in Escherichia coli
Source: ACS Synth Biol. 2023 Aug 2;12(8):2236–44. doi: 10.1021/acssynbio.3c00237 (PMC10443036; doi:10.1021/acssynbio.3c00237)
Supplement: Supplementary file 1 — sb3c00237_si_001.pdf [file sb3c00237_si_001.pdf]

Supporting Information for

Engineering chlorophyll, bacteriochlorophyll and carotenoid biosynthetic pathways in *Escherichia coli*

Guangyu E. Chen[a*] and C. Neil Hunter[b]

[a]*State Key Laboratory of Microbial Metabolism, School of Life Sciences and Biotechnology, Shanghai Jiao Tong University, Shanghai 200240, China*

[b]*School of Biosciences, University of Sheffield, Sheffield S10 2TN, United Kingdom*

*Email: guangyu.chen@sjtu.edu.cn

Table S1. Genes used to assemble (B)Chl and carotenoid biosynthetic pathways in *E. coli*.

Gene	Locus	Organism	Annotation
chlI	slr1030	*Synechocystis* sp. PCC 6803	I subunit of magnesium chelatase
chlD	slr1777	*Synechocystis* sp. PCC 6803	D subunit of magnesium chelatase
chlH	slr1055	*Synechocystis* sp. PCC 6803	H subunit of magnesium chelatase
gun4	sll0558	*Synechocystis* sp. PCC 6803	porphyrin-binding protein that enhances magnesium chelatase
chlM	slr0525	*Synechocystis* sp. PCC 6803	magnesium-protoporphyrin IX methyltransferase
bciB	slr1923	*Synechocystis* sp. PCC 6803	ferredoxin-dependent 8-vinyl reductase
chlP	sll1091	*Synechocystis* sp. PCC 6803	geranylgeranyl reductase
chlG	slr0056	*Synechocystis* sp. PCC 6803	chlorophyll *a* synthase
acsF	rge_33550	*Rubrivivax gelatinosus* IL144	O_2-dependent magnesium-protoporphyrin IX monomethyl ester cyclase
crtE	rge_33730	*Rubrivivax gelatinosus* IL144	geranylgeranyl pyrophosphate synthase
bchN	rsp_0285	*Rhodobacter sphaeroides* 2.4.1	N subunit of dark-operative protochlorophyllide oxidoreductase
bchB	rsp_0286	*Rhodobacter sphaeroides* 2.4.1	B subunit of dark-operative protochlorophyllide oxidoreductase
bchL	rsp_0288	*Rhodobacter sphaeroides* 2.4.1	L subunit of dark-operative protochlorophyllide oxidoreductase
bchC	rsp_0263	*Rhodobacter sphaeroides* 2.4.1	3-hydroxyethyl bacteriochlorophyllide dehydrogenase
bchX	rsp_0262	*Rhodobacter sphaeroides* 2.4.1	X subunit of chlorophyllide oxidoreductase
bchY	rsp_0261	*Rhodobacter sphaeroides* 2.4.1	Y subunit of chlorophyllide oxidoreductase
bchZ	rsp_0260	*Rhodobacter sphaeroides* 2.4.1	Z subunit of chlorophyllide oxidoreductase
bchF	rsp_0284	*Rhodobacter sphaeroides* 2.4.1	3-vinyl bacteriochlorophyllide hydratase
bchG	rsp_0279	*Rhodobacter sphaeroides* 2.4.1	bacteriochlorophyll *a* synthase
$crtI^{Rs}$	rsp_0271	*Rhodobacter sphaeroides* 2.4.1	3-step phytoene desaturase
$crtB^{Rs}$	rsp_0270	*Rhodobacter sphaeroides* 2.4.1	15-*cis*-phytoene synthase
dxs	b0420	*Escherichia coli*	1-deoxy-D-xylulose-5-phosphate synthase
$crtY^{Pa}$	n/a	*Pantoea agglomerans*	lycopene cyclase
$crtI^{Pa}$	n/a	*Pantoea agglomerans*	4-step phytoene desaturase
$crtB^{Pa}$	n/a	*Pantoea agglomerans*	15-*cis*-phytoene synthase

Table S2. Strains and plasmids described in this study.

Strain/Plasmid	Characteristics	Source
E. coli		
JM109	Cloning strain for plasmid construction	Promega
C43(DE3)	Expression strain for *in vivo* assay and assembly of (B)Chl and carotenoid biosynthesis pathways	Ref. 1
Synechocystis		
WT	sp. PCC 6803, glucose tolerant	R. Sobotka[†]
Rba. sphaeroides		
WT	2.4.1	S. Kaplan[‡]
Δ*bchP*	Unmarked deletion of the *bchP* gene in WT	Ref. 2
Δ*crtC*	Unmarked deletion of the *crtC* gene in WT	Ref. 3
**Plasmid**		
pET3a	Expression vector carrying T7 promoter, Amp^R	Novagen
pACYCDuet1	Expression vector with two multiple cloning sites (MCS) both preceded by a T7*lac* promoter, Cm^R	Novagen
pCDFDuet1	Expression vector with two MCS both preceded by a T7*lac* promoter, Sm^R	Novagen
pCOLADuet1	Expression vector with two MCS both preceded by a T7*lac* promoter, Km^R	Novagen
pAC-BETA	Contains *Pantoea agglomerans crtE*, *crtY*, *crtI*, and *crtB* genes with their native promoters and enables *E. coli* to produce β-carotene, Cm^R	Ref. 4
pET3a-*dvr*	*Synechocystis dvr* gene (internal *Spe*I site removed) cloned into *Nde*I/*Spe*I sites of a modified pET3a with an *Spe*I site added immediately upstream of the *Bam*HI site (same applies for all pET3a constructs described in this study), Amp^R	Ref. 5
pET3a-*chlG*	*Synechocystis chlG* gene cloned into *Nde*I/*Spe*I sites of pET3a, Amp^R	Ref. 5
pET3a-*bchCXYZ*	*Rba. sphaeroides bchCXYZ* genes cloned into *Nde*I/*Spe*I sites of pET3a, Amp^R	This study
pET3a-*bchF*	*Rba. sphaeroides bchF* genes cloned into *Nde*I/*Spe*I sites of pET3a, Amp^R	This study
pET3a-*bchG*	*Rba. sphaeroides bchG* genes cloned into *Nde*I/*Spe*I sites of pET3a, Amp^R	This study
pET3a-*bchNBL*	*Rba. sphaeroides bchNBL* genes (internal *Nde*I and *Spe*I sites removed) cloned into *Nde*I/*Spe*I sites of pET3a, Amp^R	This study
pET3a-*crtE*	*Rvi. gelatinosus crtE* gene cloned into *Nde*I/*Spe*I sites of pET3a, Amp^R	This study
pET3a-*crtYIB*	*Pantoea agglomerans crtYIB* gene fragment amplified from pAC-BETA and cloned into *Nde*I/*Spe*I sites of pET3a, Amp^R	This study
pET3a-*bchCXYZFG*	Link and lock cloning, *bchCXYZ-bchF-bchG* cloned into pET3a, Amp^R	This study
IA	Link and lock cloning, *chlI-chlD-chlH-gun4-chlM-acsF* cloned into pET3a, Amp^R	Sci. Adv.
**P1-1**	Link and lock cloning, *Synechocystis chlG* gene cloned downstream of the *acsF* gene of IA, Amp^R	This study
**P1-2**	Link and lock cloning, *Rba. sphaeroides bchNBL* genes cloned downstream of the *acsF* gene of IA, Amp^R	This study
BoP	The BoWSCP-His_{10} coding sequence and *Synechocystis chlP* gene cloned into the *Nco*I/*Hind*III sites and *Nde*I/*Xho*I sites of pACYCDuet1, Cm^R	Ref. 5
pCDFDuet1*-*dvr*	The *Xba*I–*Hind*III region containing the *lacI* gene and T7*lac* promoter 1 of pCDFDuet1 replaced with the T7 promoter–*Xba*I–*Hind*III fragment of pET3a-*dvr*, Sm^R	This study
pCDFDuet1*-*dvr*-2-*chlP*	The *Hind*III/*Xho*I fragment containing the *chlP* gene cut from BoP and cloned into pCDFDuet1*-*dvr*, Sm^R	This study
**P2-1**	Link and lock cloning, *Rba. sphaeroides bchNBL* genes cloned downstream of the *dvr* gene of pCDFDuet1*-*dvr*-2-*chlP*, Sm^R	This study
**P2-2**	The *Xba*I/*Hind*III fragment containing *bchCXYZFG* genes cut from pET3a-*bchCXYZFG* and cloned into pCDFDuet1*-*dvr* to replace the *dvr* gene, Sm^R	This study

**P2-3**	The *XbaI*/*HindIII* fragment containing *bchCXYZFG* genes cut from P2-2 and cloned into pCDFDuet1*-*dvr*-2-*chlP* to replace the *dvr* gene, Sm^R	This study
**P3-1**	The DE plasmid reported in Sci. Adv. paper with *E. coli dxs* and *Rvi. gelatinosus crtE* genes cloned into *NcoI*/*HindIII* sites and *NdeI*/*XhoI* sites of pCOLADuet1, renamed as plasmid P3-1 in this study for clarity, Km^R	Ref. 5
pCOLADuet1-*dxs*	*E. coli dxs* gene cloned at the *NcoI*/*HindIII* sites of pCOLADuet1	This study
pCOLADuet1*-*crtE*	*Rvi. gelatinosus crtE* gene with the adjacent *SpeI*–*HindIII* region of pET3a amplified from pET3a-*crtE* and cloned into *NcoI*/*HindIII* sites of pCOLADuet1, Km^R	This study
**P3-2**	*Rvi. gelatinosus crtE* gene and *Rba. sphaeroides crtIB* genes with a 42-bp RBS-containing sequence (with the last T changed to A) from pET3a added between *crtE* and *crtIB* cloned into *NdeI*/*KpnI* sites of pCOLADuet1-*dxs*, Km^R	This study
**P3-3**	Link and lock cloning, *Pantoea agglomerans crtYIB* gene fragment cloned downstream of the *crtE* gene of pCOLADuet1*-*crtE*, Km^R	This study

[*]Research Institute for Photosynthetic Hydrogen Production, Kanagawa University, Japan. [†]Institute of Microbiology, Department of Phototrophic Microorganisms, Třeboň, Czech Republic. [‡]Department of Microbiology and Molecular Genetics, The University of Texas Medical School, Houston, TX.

References

(1) Miroux, B., and Walker, J. E. (1996) Over-production of proteins in *Escherichia coli*: mutant hosts that allow synthesis of some membrane proteins and globular proteins at high levels. *J. Mol. Biol. 260*, 289–298.

(2) Hitchcock, A., Jackson, P. J., Chidgey, J. W., Dickman, M. J., Hunter, C. N., and Canniffe, D. P. (2016) Biosynthesis of chlorophyll *a* in a purple bacterial phototroph and assembly into a plant chlorophyll-protein complex. *ACS Synth. Biol. 5*, 948–954.

(3) Chi, S. C., Mothersole, D. J., Dilbeck, P., Niedzwiedzki, D. M., Zhang, H., Qian, P., Vasilev, C., Grayson, K. J., Jackson, P. J., Martin, E. C., Li, Y., Holten, D., and Hunter, C. N. (2015) Assembly of functional photosystem complexes in *Rhodobacter sphaeroides* incorporating carotenoids from the spirilloxanthin pathway. *Biochim. Biophys. Acta 1847*, 189–201.

(4) Cunningham, F. X., Pogson, B., Sun, Z., McDonald, K. A., DellaPenna, D., and Gantt, E. (1996) Functional analysis of the β and ε lycopene cyclase enzymes of Arabidopsis reveals a mechanism for control of cyclic carotenoid formation. *Plant Cell 8*, 1613–1626.

(5) Chen, G. E., Canniffe, D. P., Barnett, S. F. H., Hollingshead, S., Brindley, A. A., Vasilev, C., Bryant, D. A., and Hunter, C. N. (2018) Complete enzyme set for chlorophyll biosynthesis in *Escherichia coli*. *Sci. Adv. 4*, eaaq1407.

Table S3. Oligonucleotide primers used in this study.

Primer	Sequence (5'-3')
bchCFNdeI	GGAACATATGGTGAGAACGACCGCCGTCAT
bchZRSpeI	GGAAACTAGTTCATTGGTTCTCTCCCTTCCTCT
bchFFNdeI	GGAACATATGCAGCCCACGTCCCCCGC
bchFRSpeI	GGAAACTAGTTCATTGCGCGGCCTCCATGTC
bchGFNdeI	GGAACATATGAGTGTCAATCTATCCTTACA
bchGRSpeI	GGAAACTAGTTCACGGCAGCACCTCCAGCC
bchNFNdeI	TCTCATATGAGCCTTGACCTTCCGCC
bchNBremoveNdeIF	CTGACGCTGTGGACATACGAAGGCCCGCCCCATGTG
bchNBremoveNdeIR	CACATGGGGCGGGCCTTCGTATGTCCACAGCGTCAG
bchNBremoveHindIIIF	GCAGATCTGCCGCAAGCTCGCCAGGCCCATGGAGCG
bchNBremoveHindIIIR	CGCTCCATGGGCCTGGCGAGCTTGCGGCAGATCTGC
bchBLfusionF	AGCTCATTATGCACGGTGAGCGGACGGGACGGCAAG
bchBLfusionR	CTTGCCGTCCCGTCCGCTCACCGTGCATAATGAGCT
bchLRSpeI	TCTACTAGTTCAATCGAAACCCAGCAACTC
crtEFNdeI	TCTCATATGAACACGATGACTCGCATCGA
crtERSpeI	TCTACTAGTTCAAGCGGTCTGGGTCGGAG
crtYPaFNdeI	GGCCATATGAGGGATCTGATTTTAGTCGG
crtBPaRSpeI	GGCACTAGTCTAAACGGGACGCTGCCAAAGA
CDFDuetLLF	GCGAAGCTTGCGGCCGCATAATGC
CDFDuetLLR	GCGTCTAGAGGGAAACCGTTGTGGTCTCCCTATAGTGAGTCGTATTAGCGGTTCAGTAGAAAAGATCA
crtEFNcoI	GGCCCATGGGTAACACGATGACTCGCATCGAACA
pET3aRHindIII	GGCAAGCTTTAATGCGGTAGTTTATCAC
crtERBSR	TTGTATATCTCCTTCTTAAAGTTAAACAAAATTATTTCTAGTTCAAGCGGTCTGGGTCGGAG
RBScrtIRsF	ACTAGAAATAATTTTGTTTAACTTTAAGAAGGAGATATACAAATGCCCTCGATCTCGCCCGC
crtBRsRKpnI	GGAAGGTACCCTAGATCGGGTTGGCCCGGTT
